# Human Mesenchymal Stromal Cell (MSC) Characteristics Vary Among Laboratories When Manufactured From the Same Source Material: A Report by the Cellular Therapy Team of the Biomedical Excellence for Safer Transfusion (BEST) Collaborative

**DOI:** 10.3389/fcell.2020.00458

**Published:** 2020-06-16

**Authors:** David F. Stroncek, Ping Jin, David H. McKenna, Minoko Takanashi, Magali J. Fontaine, Shibani Pati, Richard Schäfer, Emily Peterson, Eric Benedetti, Jo-Anna Reems

**Affiliations:** ^1^Cell Processing Section, Department of Transfusion Medicine, Clinical Center, National Institutes of Health, Bethesda, MD, United States; ^2^Biomedical Excellence for Safer Transfusion (BEST), Lebanon, NH, United States; ^3^Molecular and Cellular Therapeutics, University of Minnesota, Minneapolis, MN, United States; ^4^Center for Stem Cell Biology and Regenerative Medicine, The Institute of Medical Science, The University of Tokyo, Tokyo, Japan; ^5^University of Maryland School of Medical Science, Baltimore, MD, United States; ^6^University of California, San Francisco, San Francisco, CA, United States; ^7^Institute for Transfusion Medicine and Immunohaematology, German Red Cross Blood Donor Service Baden-Württemberg-Hessen gGmbH, Goethe University Hospital, Frankfurt, Germany; ^8^Cell Therapy and Regenerative Medicine Facility, University of Utah, Salt Lake City, UT, United States

**Keywords:** mesenchymal stromal cells, bone marrow, variability, quality, transcriptome

## Abstract

**Background:**

Culture-derived mesenchymal stromal cells (MSCs) exhibit variable characteristics when manufactured using different methods and different source materials. The purpose of this study was to assess the impact on MSC characteristics when different laboratories propagated MSCs from cultures initiated with BM aliquots derived from the same donor source material.

**Methods and Methods:**

Five aliquots from each of three different BM donors were distributed to five independent laboratories. Three laboratories plated whole BM and two laboratories a mononuclear BM cell fraction. Four laboratories cultured in media supplemented with fetal bovine serum (FBS) and one laboratory used human platelet lysate (hPL). Initial cell seeding densities (i.e., P0) ranged from 19.7 × 10^3^/cm^2^–282 × 10^3^/cm^2^ and for second seeding (i.e., P1) 0.05 × 10^3^–5.1 × 10^3^ cells/cm^2^. Post-thawed MSCs from each laboratory were analyzed for cell viability, immunophenotype, tri-lineage differentiation, fibroblast colony-forming units (CFU-F), gene expression, and immunosuppressive activity.

**Results:**

Transit times from BM collection to receipt by laboratories located in the United States ranged from 16.0–30.0 h and from 41.5–71.5 h for a laboratory in Asia. Post-thaw culture derived MSCs rom BM #1, #2, and #3 exhibited viabilities that ranged from 74–92%, 61–96%, and 23–90%, respectively. CFU activity from BM #1, #2, and #3 per 200 MSCs plated averaged 45.1 ± 21.4, 49.3 ± 26.8 and 14.9 ± 13.3, respectively. No substantial differences were observed in immunophenotype, and immunosuppressive activities. Global gene expression profiles of MSCs revealed transcriptome differences due to different inter-laboratory methods and to donor source material with the center effects showing greater molecular differences than source material.

**Conclusion:**

Functional and molecular differences exist among MSCs produced by different centers even when the same BM starting material is used to initiate cultures. These results indicated that manufacturing of MSCs by five independent centers contributed more to MSC variability than did the source material of the BM used in this study. Thus, emphasizing the importance of establishing worldwide standards to propagate MSCs for clinical use.

## Introduction

MSCs are a diverse population of cells that are under investigation for the treatment of a wide range of diseases and disorders that include graft-versus-host disease (GvHD) (Le Blanc et al., 2008), stroke, (Lalu et al., 2019) Crohn’s disease (Forbes, 2017), osteogenesis imperfect (Horwitz et al., 1999), osteoarthritis, (Migliorini et al., 2019) multiple sclerosis (Uccelli et al., 2019), and cardiovascular disease (Yun and Lee, 2019). The possibility of using MSCs to treat such a wide range of conditions is likely attributable to the broad spectrum of biological effects that can be exerted by MSCs via the secretion of paracrine factors such as cytokines, chemokines, and exosomes (Phinney and Pittenger, 2017) or by MSC apoptosis (Galleu et al., 2017). Because MSCs possess these qualities, they are reported to modulate the immune response, reduce inflammation and support tissue repair by promoting cell-to-cell interactions and cellular proliferation (Phinney et al., 2015; Fontaine et al., 2016; Bagno et al., 2018).

Among the first reported successful uses of MSCs was for the treatment of a patient with severe acute GvHD (Le Blanc et al., 2004). Since then, additional reports have surfaced indicating that treatment with allogeneic MSCs can achieve complete responses or show improvement in GvHD (Le Blanc et al., 2008; Prasad et al., 2011; Ball et al., 2013). However, a recent comprehensive review of completed randomized clinical trials (RCTs) that used MSCs for the treatment of GvHD found that MSCs might have little or no effect (Fisher et al., 2019). The RCT results do not support the general conclusion that MSCs are an effective therapy for steroid-refractory acute GvHD despite previously reported positive outcomes (Fisher et al., 2019). The discrepancies found in the effectiveness of using MSCs in clinical studies may be due to the overall quality of the study design (Fisher et al., 2019), the source of MSCs, and/or to differences associated with manufacturing MSCs (Regmi et al., 2019).

At present there is no standardized protocol for culturing MSCs, but more importantly there is no acceptable potency assay for the release of MSCs for clinical therapies that predict their *in vivo* efficacy (Bieback et al., 2019). MSCs are isolated from a number of different tissue source materials (e.g., BM, adipose tissue, placental tissue, etc.) and are manufactured with different culture or preconditioning strategies (Schafer et al., 2016; Bieback et al., 2019). Some of the noted culture variables include the use of different tissue source materials for the same application, different basal medium formulations, medium supplementation, initial seeding densities, the number of passages, and the length of time MSCs are maintained in culture and frozen. Moreover, MSCs from different tissue origin as well as between BM donors may vary in gene expression, phenotype and *in vitro* function (Siegel et al., 2013; Wegmeyer et al., 2013), but the relevance of these heterogeneity generators remains unclear.

The purpose of this study was to focus on determining how much variability was associated with inter-laboratory manufacturing strategies of MSCs when the tissue source material (i.e., BM donations) used to manufacture MSCs was held constant. To address this goal, the study design was to distribute aliquots of the same tissue source material (i.e., BM collections) from three different BM donors, to five independent laboratories. Each laboratory would then use their own MSC isolation and culture strategy to manufacture and cryopreserve the MSCs. The frozen MSCs were sent to a centralized laboratory to assess their characteristics and *in vitro* function.

## Materials and Methods

### Study Design

**FIGURE 1:**
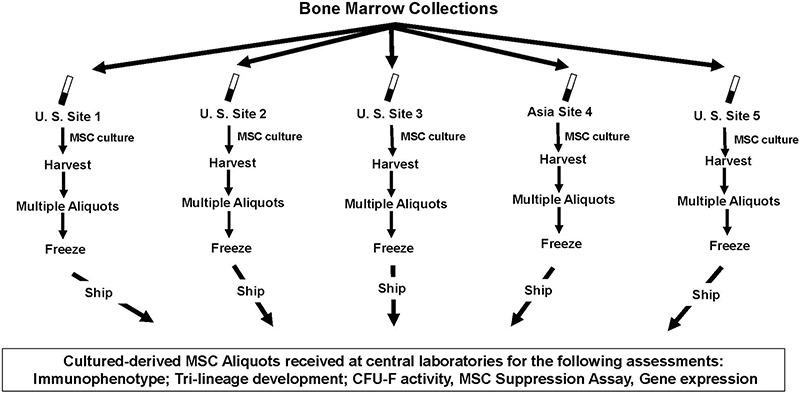
Study design. BM was collected and was divided into 10 mL aliquots (∼200 × 10^6^ MNC) and from three different donors at three different times by staff at Lonza in Walkersville, MD. Aliquots were shipped overnight to four sites located in the United States to site #4, located in Japan. Upon arrival of the BM aliquots at each site, MSC cultures were initiated according to each site’s method for culturing cells. After culturing, the MSCs were harvested, and were then cryopreserved in multiple cryovial aliquots; cryovial aliquots were frozen, and then were sent to centralized labs to perform immunophenoytping, tri-lineage differentiation potential, CFU-F quantification, gene arrays, and MSC immunomodulation.

Single 50–60 mL BM aspirates from the iliac crest were collected from three different volunteer male donors after obtaining informed consent (Lonza Walkersville, Inc., Walkersville, MD, United States) (Figure 1). BM donors #1, #2, and #3 were 21, 24, and 20 years old, respectively. The bone marrow aspirates from each of the first 2 donors, BM #1 and BM #2 were divided into 5 aliquots and BM aspirate from the 3rd donor, BM #3, was divided into six aliquots of approximately 10 mL each. Four sites (#1, #2, #3, and #4) received 1 aliquot of marrow aspirate from all 3 donors; while site #5 received 1 aliquot from BM #1 and BM #2 and 2 aliquots from BM #3. All BM aliquots were shipped to participating facilities, four of which were located within the United States and one site in Japan. FedEx conducted overnight shipments to the United States participating sites using containers with insulated packaging to maintain ambient temperature. Shipment to Japan was facilitated by MNX Global Logistics (Irvine, CA, United States) using the Evo Smart shipper (BioLife Solutions, Bothell, WA, United States) to maintain ambient temperature. Upon arrival of the BM aspirate, each site plated the BM using their own methodology to produce MSC preparations in culture.

### MSC Expansion and Cryopreservation

Cell viability and cell count assessments, culture expansions and cryopreservation were performed according to each individual sites preferred method (Tables 1–3). For all three BM aspirates, two of the sites (site #2 and #3) plated cells from whole BM and two sites (site #1 and #4) plated cells from a mononuclear cell (MNC) fraction (Table 2). Site #5 plated whole BM for all three BM aspirates and received an additional BM aliquot from BM donor #3, which they plated cells from a MNC fraction (Table 2). Initial and subsequent passage seeding densities are indicated in Table 3. The adherent cells were cultured according to each sites’ in-house protocol and upon reaching confluence; the cells were harvested and passaged as indicated in Tables 2, 3. Four sites used fetal bovine serum (FBS) as media supplement, and one site used hPL (Cell Xpand^TM^, University of Utah) (Table 2). At the end of each passage, cell counts and viabilities were performed and the number of population doublings (PDs) were calculated according to the following equation: PD(hrs) = *t* log 2/(log Nf/Ni), where PD = population doubling, t = time in culture, Nf = number of cells harvested, Ni = number of cells seeded. At the end of the culture period, each site prepared multiple aliquots of MSCs and used their own cryopreservation method to freeze the cultured-derived MSCs at a concentration that ranged from 1.0 × 10^6^ to 10 × 10^6^ cells/mL (Table 4). Laboratories routinely tested for bacterial and mycoplasma contamination. Cryopreserved cells were maintained in LN2 storage until they were shipped in LN2 to centralized testing laboratories where they were analyzed for immunophenotype, tri-lineage differentiation potential, CFU-F activity, gene expression, and immunosuppressive activity.

**TABLE 1:** Cell counting methods used by each site.

	Site #1		Site #2		Site #3		Site #4		Site #5	
	Cell Count	Viability	Cell Count	Viability	Cell Count	Viability	Cell Count	Viability	Cell Count	Viability
Whole BM	Automated (SysmexXS-1000i)	Fluorescent microscope (AO/PI)	Automated (Ad via 1200)	Flow Cytometry (7-AAD)	Manual (hemacytometer)	Light microscope (trypan blue)	Manual (hemacytometer)	Light microscope (trypan blue)	Automated (sysmexXE-5000)	Cellometer (AO/PI)
Passaged Cells	Automated (Sysmex XS-1000i)	Fluorescent microscope (AO/PI)	Cellometer (AO/PI)	Cellometer (AO/PI)	Manual (hemacytometer)	Light microscope (trypan blue)	Manual (hemacytometer)	Light microscope (trypan blue)	Manual (hemacytometer)	Light microscope (trypan blue)

**TABLE 2:** Comparison of MSC culture components used by each of the five sites.

Components	Site #1	Site #2	Site #3	Site #4	Site #5
Cells plated	MNC	Whole BM	Whole BM	MNC	Whole BM or MNC
Basal medium	αMEM	αMEM	αMEM	MEM-Eagle	DMEM
Serum supplement	16% FBS	20% FBS	10% FBS	16.5% FBS	10% PL-S
Additional supplements	GlutaMAX	None	L-glutamine	L-glutamine	GlutaMAX
Antibiotics	None	Gentamicin	Pen/Strep	Pen/Strep	Pen/Strep

*MNC, ficoll preparation; aMEM, minimum essential medium eagle alpha modification; DMEM, dulbecco’s modified eagle medium; FBS, fetal bovine serum; PL-S, serum based platelet lysate; glutaMAX an alternative to L-glutamine; Pen/Strep, penicillin streptomycin.*

**TABLE 3:** Plating densities for each marrow preparation by site and passage.

Culture vessel type and passage number	Number of cells plated/cm^2^ (Average ± SD)				
	*Site #1	*Site #2	*Site #3	*Site #4	*Site #5
Culture vessel type	Cell stack	Flasks	Flasks	15 cm dishes	Flasks
Initial seeding to obtain P0 MSCs	136,667 ± 23,094	282,000 ± 5,292	200,407 ± 43,808	19,737 ± 0	240,478 ± 38,708
Second seeding of P0 MSCs to obtain PI MSCs	50 ± 1	3,165 ± 1	2,500 ± 707	2,758 ± 4,425	2,776 ± 1,807
Third seeding of P1 MSCs to obtain P2 MSCs		3,165 ± 1	*4,055		**2,336 ± 232
Fourth seeding of P2 MSCs to obtain P3 MSCs		3,164 ± 1			***2,000

*Average ± Std. Dev. *n* = 3; **n = 2; ***n = 1.

**TABLE 4:** Cryopreservations methods used by each site.

	Site #1	Site #2	Site #3	Site #4	Site #55
Cell concentration	1–10 × 10^6^ Cells/mL	1–10 × 10^6^ Cells/mL	1.5–5 × 10^6^ Cells/mL	5 × 10^6^ Cells/mL	1–5 × 10^6^ Cells/mL
Final Freezing Solution	30% Plasmalyte-A, 10% DMSO, 2.5% Human serum albumin	Plasmalyte A, 6% Pentastarch, 5% DMSO, 2%, Human serum albumin	60% D-MEM, 30% Hyclone FBS, 10% DMSO	MEM-Eagle 15% FBS, 10% DMSO	90% FBS, 10% DMSO
Freezing Method	Control Rate Freezer	Mr. Frosty Freezing Container	Mr. Frosty Freezing Container	No Container	Mr. Frosty Freezing Container
	Start Chamber = 0.0°C;	−80°C O/N; transfer	−80°C O/N; transfer	−80°C O/N; maintain	−80°C O/N; transfer
	Sample = 1.0°C	to LN2 freezer	to LN2 freezer	in −80°C freezer	to LN2 freezer
	Ramp 1.0°C/min until				
	Sample = –12.0°C				
	Ramp 20.0°C/min until				
	Chamber = –60.0°C				
	Ramp 15.0°C/min until				
	Chamber = –18.0°C				
	Ramp 1.0°C/min until				
	Sample = –60.0°C				
	Ramp 3.0°C/min until				
	Sample = –100.0°C				
	End and transfer to LN2				
	Freezer				
Storage freezer	LN2	LN2	LN2	−80°C	LN2

### Immunophentyping

Evaluation of post-thaw MSCs generated in culture at each of the 5 sites was conducted by a centralized facility using flow cytometry to determine the expression or lack of expression of surface markers for CD105, CD73, CD90, CD45, CD14, CD34, HLA-DR, and Stro-1. Approximately 250,000 cells were transferred into each of five separate tubes. Cells from one tube were stained with primary antibodies, anti-CD73 conjugated to allophycocyanin (APC) (BD Biosciences), anti-CD90 fluorescein isothiocyanate (FITC) (BD Biosciences) and anti-CD105 phycoerythrin (PE) (Miltenyi Biotec). In a second tube, cells were triple stained with anti-CD45, anti-CD34, and anti-CD14 that were all conjugated with PE-Cy^TM^5 (BD Biosciences). In a third tube cells were stained with anti-Stro1 APC (BioLegend) and anti-HLA-DR FITC (BD Biosciences). Additional tubes were stained with isotype controls and one tube was left unstained as a control. All tubes included the use of 7-aminoactinomycin D (7-AAD) to evaluate cells for viability. Following staining, the cells were washed with PBS-BSA, resuspended in PBS-BSA, and were analyzed on a BD FACSCanto (BD Biosciences) flow cytometer.

### Tri-Lineage Differentiation Potential

Adipogenesis, osteogenesis, and chondrogenesis differentiation *in vitro* assays were performed by a centralized laboratory using commercially available StemPro^TM^ kit adipogenesis, StemPro^TM^ kit chondrogenesis and Gibco^TM^ kit osteogenesis as per the manufacturer’s differentiation assay protocols. Briefly, cells from each MSC preparation provided by each site were washed and resuspended using Gibco^TM^ MesenPro RS medium (ThermoFisher Scientific). Cells from each MSC preparation were then seeded into four wells (one control well to remain undifferentiated, plus three conditional replicates) of a 12-well tissue-culture plate. Adipogenic (and control) wells were seeded at 1 × 10^4^ cells/cm^2^. Osteogenic (and control) wells were seeded at 5 × 10^3^ cells/cm^2^. MSC cultures were incubated at 37°C in a humidified atmosphere of 5% CO_2_ for 48–50 h, then MesenPro RS medium in the triplicate conditional wells was removed and replaced with either adipogenesis or osteogenesis differentiation medium (StemPro^TM^ kit). The cultures were continued for 12 (adipogenic) or 18–20 (osteogenic) more days. Differentiation medium was exchanged every 2–3 days. Control wells received MesenPro RS media exchanges concurrently as the conditional wells received osteogenesis or adipogenesis media exchanges.

Chondrogenic (and control) wells were each seeded with five “micromass” 5-μL droplets of cells concentrated at 1.6 × 10^7^ cells/mL. Cells were maintained at 37°C in a humidified atmosphere of 5% CO_2_ for two hours, after which the StemPro^TM^ Differentiation kit chondrogenesis medium (ThermoFisher Scientific) was added to the triplicate conditional wells and MesenPro RS medium to the controls. Micromass/medium cultures were maintained at 37°C/5% CO_2_ for 21 days. Differentiation medium was exchanged every 2–3 days. Control wells received MesenPro RS media exchanges concurrently with the conditional wells receiving osteogenic or adipogenic media exchange. Control wells received MesenPro RS media exchanges concurrently as the conditional wells received chondrogenesis media exchanges.

Control and differentiated cell cultures were fixed with 10% formalin, and then stained with Oil Red O (adipogenesis), Alizarin Red S (osteogenesis) or Alcian Blue (chondrogenesis) dyes (all from Sigma-Aldrich). Differentiation of cells (or lack thereof) was then scored by three different staff members via visualization under an inverted light microscope from zero to four plus. A score of zero was equal to no differentiation while a score of 4+ was equal to maximum differentiation. A final score was determined as an average of the three scores as assigned by the observers.

### Fibroblast Colony-Forming Unit Assay

To determine relative Colony-Forming Unit (CFU-F) activity of for each MSC preparation, a centralized facility received frozen cultured-derived MSCs from each of the five sites derived from BM#1, BM#2, and BM#3. The centralized laboratory thawed and plated the MSCs in triplicate into 6 well plates. Each well was plated with 200 MSCs in a total of 2 mL of MesenCult MSC Basal Medium (StemCell Technologies, Vancouver, Canada). The cells were cultured for 14 days at 37°C with 5% CO_2_. After 14 days, the media form each well was aspirated and adherent cells were washed with 2 mL PBS, then 2 mL ice-cold methanol was added to each well for 5 min to fix the cells. The methanol was aspirated and 2 mL Wright-Giemsa Stain (Sigma Aldrich, St. Louis, MO, United States) was added to each well and stained for 10 min. The stain was aspirated and the wells washed twice with 2 mL PBS. The plates were air-dried and colonies were counted using a light microscope.

### Mixed Lymphocyte Reaction

The immunosuppressive properties of MSCs were compared using MLR assays (SAIC-Frederic, Frederic, MD, United States). Ficoll-separated peripheral blood mononuclear cells were plated in 96-well plates at 1 × 10^5^ responders per well. Responders were co-cultured with 2500 cGy irradiated stimulator peripheral blood mononuclear cells at a concentration of 1 × 10^5^ cells per well. MSCs from different centers were added at concentrations of 1 × 10^4^, 4 × 10^4^, and 10 × 10^4^ cells/well. Culture plates were incubated for 6 days in a humidified 5% CO_2_ incubator at 37°C. On the day of harvest, 0.5 μ Ci of ^3^H-thymidine was added to each well for 4 h with lymphocyte proliferation measured using a liquid scintillation counter. The effect of MSCs on MLR was calculated as the percentage of the suppression compared with the proliferative response of the control without MSCs, where the control was set to 0% suppression. The experiments were performed three times for each variable described.

### Global Gene Expression Analysis Using Microarrays

Total RNA extractions were performed on the samples from each of the different centers using RNeasy Mini Kit (Qiagen) according to the manufacturer’s protocol. RNA was quantified using Nanodrop 8000 (Thermo Fisher Scientific, Wilmington, DE, United States). Total RNA integrity was evaluated following isolation using a 2100 Bioanalyzer (Agilent Technologies, Santa Clara, CA, United States). Samples with an RNA Integrity Number value ≥8 were used for gene expression analysis.

Microarray gene expression analysis was performed on 4 × 44 K Whole Human Genome Microarrays (Agilent Technologies, Santa Clara, CA, United States) according to the manufacturer’s protocol. In general, 200ng of total RNA from each sample was amplified, labeled, and hybridized on the array chip using a Quick Amp Labeling kit (Agilent). Array images were obtained by Agilent Scanner G2600D. Then images were extracted using Feature Extraction 12.0 software (Agilent). Partek Genomic Suite 6.4 (Partek Inc., St. Louis, MO, United States) was used for data visualization and hierarchical cluster analysis.

### Statistical and Microarray Data Analysis

Principal Component Analysis (PCA) was used to visualize the similarities and differences among the samples from different centers. Unsupervised Hierarchical clustering was performed by Partek Genomic Suite using whole gene set to group similar samples into clusters.

The Source of Variation Analysis was performed to present the relative contribution of each factor included in the ANOVA by Partek Genomic Suite and the *F*-value provided by this analysis is the ratio of between group variance/within group variance. A large *F*-value for a factor indicates that between group variation is greater than within group variation.

## Results

### Bone Marrow Collection and Distribution

BM aspirates (i.e., 50–60 mL each) were collected from three different volunteer donors and the BM from each donor was divided into aliquots of approximately 10 mL each (Figure 1). The BM aliquots were shipped to 4 sites located in the United States and to 1 site located in Japan. The average time from the collection to the receipt of the BM at each site is shown in Table 5. For those samples transported within the United States the average transit time was approximately 24 h while the average shipment time to the site located in Japan was approximately twice as long. Upon arrival at each site, the initiation of processing was executed within 5 h of receipt of the BM sample (Table 5).

**TABLE 5:** Average shipping time for marrow collection to manufacturing site and time for receipt of marrow to the initiation of processing.

	Shipping		Receipt to processing	
Site#	time (hrs)		processing time (hrs)	
	Average	Range	Average	Range
1	26.8	25.0–27.0	4.3	3.0–6.0
2	28.0	26.5–30.0	3.7	3.0–5.0
3	22.4	16.0–25.8	4.3	2.8–5.0
*4	55.2	41.5–71.5	1.5	0.0–3.5
5	26.2	25.0–27.0	4.3	3.0–6.0

*lnternational.

### Inter-Laboratory Method Comparisons for Producing MSCs in Culture

**FIGURE 2:**
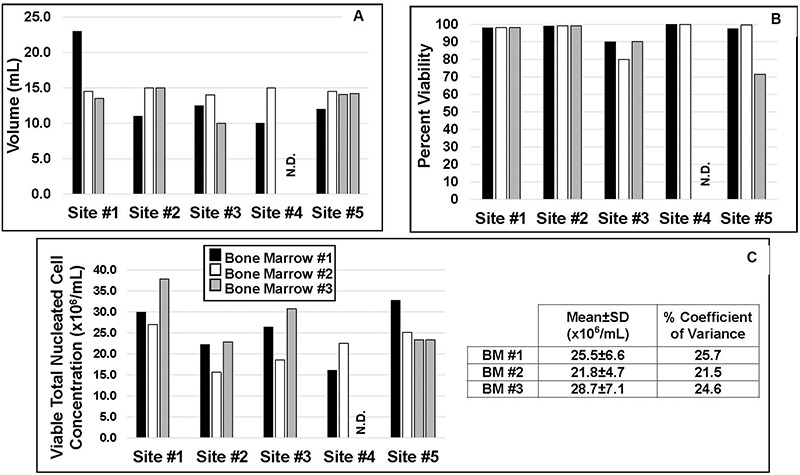
Characteristics of each of three BM aliquots as evaluated by the respective manufacturing site. **(A)** The volume of BM aspirate received at each site. **(B)** Percent viability of nucleated cells. **(C)** The average concentration of viable total nucleated cells from each of the three donors. The table shows the inter-laboratory Coefficient of Variance for cell concentrations. ND = not done.

Each site evaluated the BM aliquots for volume, cell viability, and total number of nucleated cells (Figure 2). The requested minimum volume from each of three donors to be supplied by the procurement agency to each site was 10 mL. As shown in Figure 2A, all sites received a minimum of 10 mL from each donor with some sites receiving volumes of greater than 10 mL. Cell viabilities as performed by each site (Table 1) were typically 98–100%, with one site, site #3, reporting viabilities from 80–90%, and one site, site #5, reporting that one of its aliquots had a viability of approximately 71% (Figure 2B). Average viable TNC concentrations for BM aspirates #1, #2, and #3 from the 5 laboratories were 25.5 ± 6.6 × 10^6^/mL, 21.8 ± 4.1 × 10^6^/mL, and 28.7 ± 7.1 × 10^6^/mL with coefficients of variation of 25.7, 21.5, and 24.6%, respectively (Figure 2C).

**FIGURE 3:**
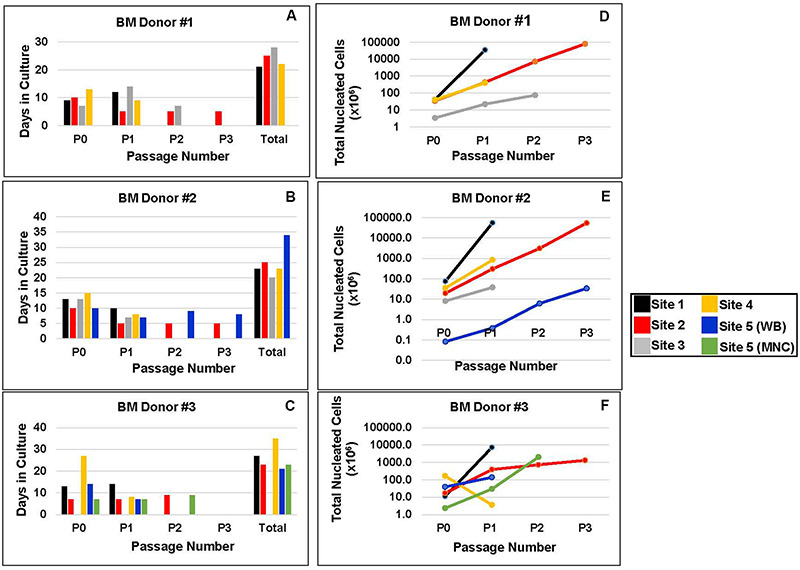
MSC expansion. **(A–C)** The number of passages and the time in culture for each marrow sample as performed by each site. **(D–F)** The theoretical number of adherent cells obtained at the indicated passage by each manufacturing site from each marrow sample. P0 = cell numbers after initial seeding; P1 = cell counts after 2nd seeding; P2 = cell counts after 3rd seeding; and P3 = cell counts after 4th seeding. Since sites did not seed all cells to obtain passages 1, 2, and 3, theoretical cell yields were calculated to predict the number of cells that would have been obtained if all cells had been plated from the previous passage. This was accomplished by first dividing the number of cells harvested by the number of cells seeded for its subsequent passage and then multiplying the actual cell yields obtained for P1, P2, and P3. For example: P1 theoretical cell yields = (P0_ay_/P1_as_)xP1_ay_. P0_ay_ = actual yield of cells at P0; P1_as_ = actual number of cells seeded to obtain passage 1 cells; P1_ay_ = actual yield of cells at P1.

The culture schemes used by each of the five centers are summarized in Tables 2, 3. Two sites used unfractionated whole BM to plate cells, two sites subjected the BM aspirate to a density gradient separation to plate cells from a mononuclear cell fraction (MNC), and one site used either whole marrow or a MNC cell fraction to initiate MSC cultures. Laboratories cultured cells in alpha minimal essential media (MEM), MEM-Eagle or Dulbecco’s Modified Eagle Medium (DMEM). Fetal bovine serum (FBS) was used by 4 facilities as a media supplement, and 1 laboratory used a commercially available human platelet lysate-serum (PL-S) (Cell Xpand, University of Utah). The final concentration of FBS ranged from 10% to 20% and the final concentration for PL-S was 10% (Table 2). Antibiotics were added to the medium by four of the five centers (Table 2). Three sites cultured the cells in T-flasks, one site in a multiple layer flask and one site in dishes of 15 cm diameter (Table 2). Primary cultures or the initial seeding cell densities for BM#1, BM#2, and BM#3 ranged from 0.2 × 10^5^ cells/cm^2^ to 2.8 × 10^5^ cells/cm^2^ (Table 3). After the initial seeding, the five sites inoculated MSCs from the primary cultures (i.e., P0) at seeding densities that ranged from 50 cells/cm^2^ to 4.0 × 10^3^ cells/cm^2^ to obtain P1 MSCs. After harvesting MSCs at P1, sites 1 and 4 cryopreserved their cells without any further culturing. Three sites sub-cultured P1 MSCs at seeding densities that ranged from 2.3 × 10^3^ cells/cm^2^ to 4.0 × 10^3^ cells/cm^2^ to obtain MSCs from a P2 harvest (P2) (Table 3). After a P2 harvest, site 3 cryopreserved their cells without any further culturing. Two sites inoculated cultures with P2 MSCs to obtain MSCs that were cryopreserved after passage 3 (P3). No site passaged MSCs beyond P3 (Table 3). This translated into overall culture periods that ranged from 21 to 28 days for BM #1, 20 to 34 days for BM #2, and 21 to 35 days for BM #3 (Figures 3A–C). We did not only observe inter-laboratory variances for the time cells spent in culture at a particular passage among the five laboratories, but there were also intra-laboratory differences in the amount of time that the MSC cultures from different BM donors spent at a specific passage (Figures 3A–C). Each site cryopreserved cells according to their in-house protocol (Table 4). Four of the sites used 10% DMSO and one site used 5% DMSO as the final concentration of cryoprotectant. Only one site used a control rate freezer to drop the cyrovial temperature to −100°C, and three sites used a Thermo Scientific Nagene Mr. Frosty Freezing container to drop the temperature to −80°C before transferring to a liquid nitrogen freezer. One site wrapped the cryovials and placed them at −80°C and maintained them at this temperature for shipment to the centralized testing facilities.

In the end, different numbers of passages were executed by the five sites for each of the BM donors. For BM #1, the final harvest of cells for two sites occurred at P1, 1 site at P3 and one site failed to obtain cells. For BM#2, the final harvesting of cells occurred for three of the sites at P1 and for two sites at P3. Finally, for BM#3, three sites harvested at P1, one site at P2 and one site at P3. Overall, this resulted in 14 different final preparations of MSCs that were frozen according to each sites’ own specific freezing protocol for maintenance in LN2 prior to their analysis for phenotype and function. In summary, among the five laboratories the variations in methodologies to produce MSCs in culture included: (1) how cells were plated (i.e., cell density, whole BM versus MNC preparations); (2) the culture medium, (3) the time in culture; (8) the number of passages performed before the final cell harvest was cryopreserved and; (9) how each laboratory cryopreserved their cells.

### Proliferation Responses

**FIGURE 4:**
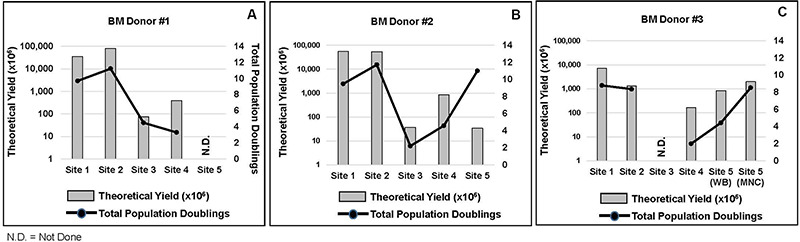
MSC yields and population doublings. For each BM aspirate and site the theoretical MSC yields and population doubling (PD) are shown. **(A)** Shows the results for BM donor #1, **(B)** the results for BM donor #2 and **(C)** for BM donor #3.

Cellular proliferation responses as measured by theoretical yield and population doubling time from 14 different seeding events from three BM donors from five laboratories are shown in Figures 3D–F4A–C. Overall, inter-laboratory differences in cell proliferation responses and cell yields were observed from each BM donor. The most notable observations include the following: (1) Cell yields for all three BM donors showed that site #1 consistently produced the most cells and this was accomplished with only a P1 harvest. (2) Site #2 reported cell yields from BM #1 and BM #2 that were comparable to those obtained by site #1, but this was only achieved after site #2 completed a P3 harvest (Figures 3D,E4A,B). (3) Cell cultures for BM #2 performed by site #5 showed that the cellular proliferation responses lagged relative to the other four sites (Figures 3E 4B). (4) In general proliferation responses from the MSCs isolated from BM #3 were muted relative to the proliferation responses for cells from BM #1 and BM #2 (compare Figure 3F to Figures 3D,E and Figure 4C to Figures 4A,B).

### Evaluation of MSCs Post-thaw

To determine whether inter-laboratory manufacturing differences affected the phenotype and function of the cultured MSCs after cryopreservation, the frozen MSCs from 14 final preparations or lots were thawed and examined for cell surface markers, cell viability, CFU-F activity, tri-lineage differentiation potential, immunosuppressive activity, and their gene expression profile. This meant that assays were performed on 14 lots of MSCs that were cryopreserved at different passages during the manufacturing process. Sites #1 and #4 provided 6 lots of P1 MSCs and site #2 provided 3 lots of P3 MSCs from each of the 3 BM donors. Site #3 provided 2 lots of P2 MSCs from BM#1 and BM#2 donors. In addition, site #5 provided 1 lot of P3 MSCs from BM donor #2, and 1 lot of P1 and 1 lot of P2 MSCs from BM donor #3.

**FIGURE 5:**
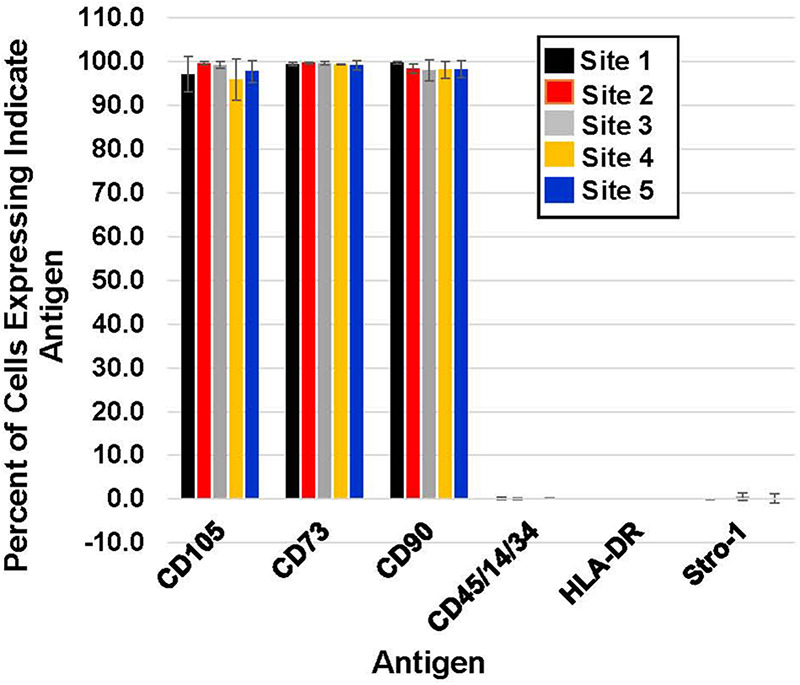
Immunophenotyping of MSCs. 14 lots of cryopreserved MSCs from the five laboratories were analyzed by flow cytometry at a single laboratory. The MSCs were analyzed for the expression of CD105, CD73, CD90, CD45/CD14/CD34, HLA-DR and Stro-1 following thawing.

To assess whether each of the 14 final preparations of MSCs met the criteria established for a MSC phenotype, each lot of cells was examined by flow cytometry for their expression of cell surface antigens CD105, CD73, CD90 and lack of express for CD45/14/34 and HLA-DR) (Dominici et al., 2006). All final harvests of cells expressed the antigens CD105, CD73, and CD90 and lacked the expression of CD45/14/34 and HLA-DR as well as the Stro-1 antigen without apparent inter-laboratory differences (Figure 5).

**FIGURE 6:**
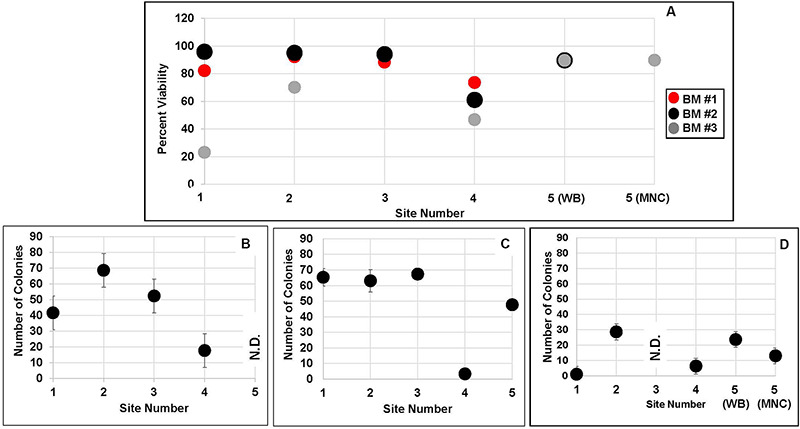
MSC post-thaw viability and CFU activity. **(A)** Percent viabilities for all MSCs produced from the three donors and by the five sites and **(B)** the absolute number of CFU-F colonies produced by MSCs derived at each of five sites from cultures that were initiated with aliquots from **(B)** BM donor #1; **(C)** BM donor #2; and **(D)** BM donor #3. ND = not done.

**FIGURE 7:**
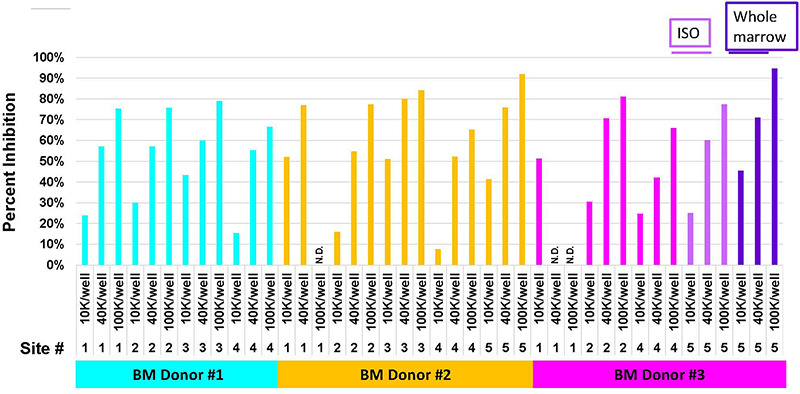
Mixed-Lymphocyte-Reaction of MSCs manufactured from the same three donors at five manufacturing sites. MLR data shows inhibition of lymphocyte proliferation when the BM-derived MSCs were added and the inhibition increased as the ratio of MSCs to lymphocytes increased. Results from three donors and five centers are shown. For some samples no data is shown since not enough MSCs were available to test.

Next, we examined each of the 14 lots of MSCs to determine whether inter-laboratory differences in culture strategies affected their function. Post-thaw MSC viabilities ranged from 74 to 92% for BM#1, from 61 to 96% for BM#2 and from 23–90% for BM#3 (Figure 6A). Site #4 had the lowest viabilities and the poorest CFU-activity for MSCs produced from BM donors #1 and #2. While site #1 had the lowest MSC viabilities and CFU-F activity for BM donor #3. Overall, MSC viabilities and CFU-F activity from BM#3 tended to be lower than for MSCs from BM donors #1 and #2 (Figures 6B–D). Inconsistencies in tri-lineage development among the laboratories were also observed and again site#4 showed some of the poorest developmental activity (Table 6). Of note is the fact that site#4, which was a laboratory located in Japan, experienced the longest transport times from BM collection to culture initiation and also shipped their cells at −80°C. Also, greater inconsistencies in tri-lineage development were observed from MSC lots produced from BM#3 than from MSCs isolated from the other two BM donors (Table 6). Interestingly, despite poorer viabilities, CFU-F activity and tri-lineage differentiation potential for MSCs from BM#3, the immunosuppressive activity of MSCs from BM#3 were comparable to that of MSCs from BM#1 and BM#2 (Figure 7).

**TABLE 6:** Tri-lineage potential of post-thaw MSCs by manufacturing site.

Adipogenesis				Chrondrogenesis				Osteogenesis			
Site number	Prep #1	Prep #2	Prep #3	Site number	Prep #1	Prep #2	Prep #3	Site number	Prep #1	Prep #2	Prep #3
	*Average	*Average	*Average		*Average	*Average	*Average				
Site l	2.0	2.3	0.5	Site l	3.0	3.0	0.5	Site l	Pos	Pos	poor diff
Site 2	3.2	4.0	4.0	Site 2	3.0	3.0	3.0	Site 2	Pos	Pos	Pos
Site 3	2.0	4.0	N.D.	Site 3	3.0	3.0	N.D.	Site 3	Pos	Pos	Pos
Site 4	1.0	0.8	0.8	Site 4	3.0	0.0	1.3	Site 4	Pos	Pos	Pos
Site 5 WB	N.D	3.0	3.0	Site 5 WB	N.D.	3.0	3.0	Site 5 WB	N.D.	Pos	Pos
Site 5 MNC	N.D.	N.D.	2.0	Site 5 MNC	N.D.	N.D.	3.0	Site 5 MNC	N.D.	N.D.	Pos
Average	2.0	2.8	2.1	Average	3.0	2.4	2.0				

Scale 0–4; where 0 = no differentiation and 4 = maximum differentiation. N.D. = Not Done. *n = 3.

### Global Gene Expression Analysis

**FIGURE 8:**
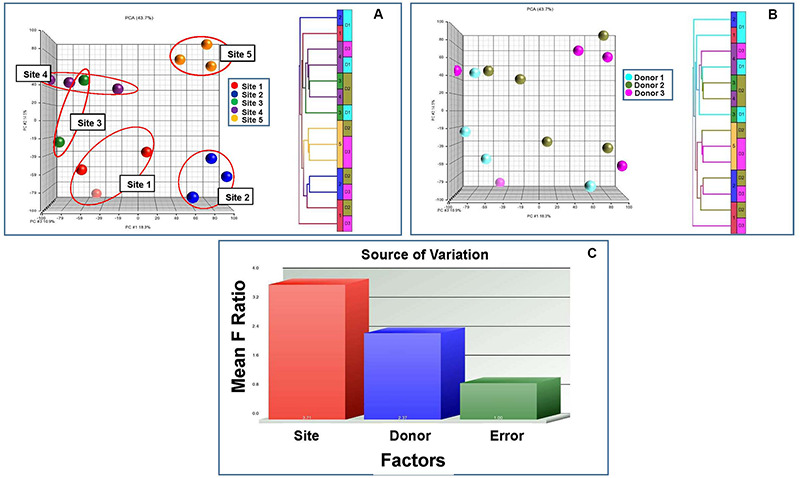
Principle Component Analysis (PCA), unsupervised hierarchical clustering analysis and Source of Variation Analysis of gene expression data from MSCs manufactured from the same three donors by five different manufacturing sites. PCA and unsupervised hierarchical clustering analysis shows that there are differences in MSC transcriptomes due to the manufacturing laboratory and to donor variability. **(A)** The results of PCA analysis for gene expression data of MSC lots are color coded by manufacturing site, **(B)** PCA analysis for gene expression of MSCs are color coded by donor source and **(C)** unsupervised hierarchical clustering analysis. Source of Variation Analysis shows greater transcriptome variation due to laboratory methodology than with donor BM source.

All 14 lots of MSCs were analyzed by global gene expression analysis including PCA and unsupervised hierarchical analysis by using the entire set of expressed genes to identify relationships among the samples. Gene expression of MSC lots clustered by manufacturing site and donors (Figures 8A,B). Notably, the MSC lots clustered stronger according to site compared to donors (Figures 8A,B). This was confirmed by unsupervised hierarchical clustering analysis showing that the grouping of samples was related to site and donor with sites having a greater effect (Figure 8C). To further evaluate the relationship between the effects of manufacturing site and donor variability on MSC characteristics, the entire set of expressed genes from the 14 MSC lots was subjected to source of variation analysis. This analysis confirmed that the variability among MSCs samples was due to both the site and the BM donor, but the manufacturing site had a greater influence on MSC characteristics (Figure 8C).

## Discussion

Manufacturing MSCs of consistent quality and potency is important for their utilization as effective clinical therapeutics. For the purpose of this discussion the MSC manufacturing process is divided into the four following steps: (1) Donor selection and BM collection; (2) maintenance and transport of the BM aspirate from the collection site to the processing site; (3) culture strategy (i.e., plating cells, passaging the adherent cells and harvesting the MSCs); and (4) cryopreservation and storage of the manufactured MSCs.

Previously, we reported that MSCs exhibited variable characteristics and functions when multiple participating sites used different tissue sources as well as different manufacturing methods (Liu et al., 2017). Herein, we report that inter-laboratory manufacturing differences make a greater contribution to MSC variability than the differences noted among the BM donors utilized in this study. An observation that suggests that standardizing the manufacturing process of culture-derived MSCs from BM aspirates may lead to reduced variability in MSC characteristics and functions.

Evidence is presented in this study that there are inter-donor differences. Specifically, MSCs from donor #3 were found to be less proliferative than those from donors #1 and #2 by all sites and formed less CFU-F by four of the five sites. Such variable growth kinetics of MSC preparations have been reported as being attributable to donor-related variables, such as age and sex (Siegel et al., 2013; Detela et al., 2018). This is also highlighted in studies reporting that BM-MSCs from younger donors have distinct molecular signatures and feature superior wound healing efficacy in a mouse model (Siegel et al., 2013; Khong et al., 2019). Although this is a small study, it is unlikely that sex or age is a major contributing factor for the lower proliferative responses from donor #3 as compared to donors #1 and #2 BM aspirates. All of the donors are males and the ages of BM donor #1, #2, and #3 are 21, 24, and 20 years old, respectively.

Manufacturing concepts of MSC therapies vary worldwide with variability in MSC culture strategy and product release specifications (Trento et al., 2018). Consequently, it is not surprising that the inter-laboratory differences of this study, which reflect manufacturing variabilities, resulted in MSCs with variable proliferation rates, total expansion numbers, CFU-F content and MSC tri-lineage differentiation potential. With regard to surface marker expression, we did not find substantial differences among sites. Yet, we tested only for “binaric” (i.e., either high or very low expressed) MSC markers, and not for markers that define MSC subpopulations such as CD271 or CD146 (Tormin et al., 2011; Kuci et al., 2019). Higher resolution analyses quantifying MSC subpopulations might have revealed an impact of the manufacturing strategies on MSC subpopulation compositions of the products. Another limitation of our study is that not all sites seeded all the available cells from a harvest to produce cells for a subsequent passage. Therefore, theoretical cell yields were calculated to predict the number of cells that would have been obtained if all cells had been plated from the previous passage.

MSCs produced by each site were also evaluated by global gene expression analysis. Differences in gene expression were found that could be attributed to both variability among BM donors and manufacturing site. While the sample size of the study was not large enough to assess the nature of these differences, the analysis showed that the inter-laboratory differences made a greater contribution to MSC variability than did differences among BM donors. This aligns with a recent study reporting that variable *in vitro* expansion strategies (i.e., passaging or “*in vitro* aging”) have a stronger impact on MSC molecular phenotype than donor age (Andrzejewska et al., 2019).

There are a number of differences in laboratory practices that likely contributed to the MSC variabilities observed in this study. One such variance in inter-laboratory practices is transport time for the BM harvest to the processing site. Although all BM aliquots were transported at ambient temperatures, transport times at one site varied substantially. Transit times to the four sites located in the United States ranged from 16 to 30 h while for site #4, located in Japan, transit times ranged from 41 to 71 h. Coincidently, the lowest CFU-F content of the manufactured MSCs occurred at site #4. Granting that this is a small data set, if a maintenance temperature of 20–25°C is used, these results indicate that a BM aspirate should probably be processed within 24–36 h of collection. Also, of note is the observation that proliferation capacities and yields of MSCs were greater for sites #1 and #2, which may have been due in part to the use of higher concentrations of FBS as a culture medium supplement by site #1 (16%) and site #2 (20%). Likewise, total population doublings of MSCs cultured using hPL at site #5 were in a higher range as compared to MSC produced by the other sites using FBS. Since hPL is shown to impact MSC biology beyond proliferation, such as in their differentiation potential and immunomodulation capacity (Menard et al., 2013; Bieback et al., 2019). It is likely that the use of hPL is another practice that contributed to MSC variability is the use of hPL by only one site in our study. Other differences in inter-laboratory practices that are associated with culture strategy are likely to have also contributed to MSC variability along with differences in inter-laboratory practices for cryopreservation and storage of MSCs.

These results support the idea that it may be possible to reduce MSC variabilities associated with inconsistencies in inter-laboratory manufacturing practices. Some steps of the manufacturing process may be more amendable to standardization and while others may not be. For instance, since MSCs are used for a number of different clinical applications, a manufacturing process that works the best for one therapy may not produce MSCs with functional qualities that are best for other therapies. Consequently, premature establishment of standards based on a culture strategy for one specific clinical application (i.e., basal medium formulations, medium supplementation, initial seeding densities, the number of passages, and length of time MSCs are maintained in culture) could end up having detrimental consequences for other applications. Conversely, standardizing inter-laboratory practices for manufacturing MSC that are not dependent on intended clinical applications (i.e., donor selection, BM collection, maintenance and transport of the BM aspirate from the collection site to the processing site, cryopreservation and storage) are urgently needed. For example, it would be advantageous for the MSC field to promote the use of superior methods like automated cell counts and the use of fluorescent dyes for viability testing over that of manual cell counts and the use of trypan blue for viability assessments to characterize whole BM aspirates prior to culture initiation (Mascotti et al., 2000).

In the end, the results of this study indicate that even if inter-laboratory manufacturing is standardized, differences among MSC preparations may still exist due to differences in the donor source of cells used for manufacturing MSCs. To build off of this study for the purpose of acquiring more information that may lead to future recommendations about standardizing MSC manufacturing, the BEST collaborative is currently planning a follow-up inter-laboratory study, in which laboratories will use the same source material and the same manufacturing, cryopreservation and storage protocols.

## Data Availability Statement

This article contains previously unpublished data. The name of the repository and accession number(s) are not available.

## Author Contributions

DS and J-AR are responsible for experimental design, lab participation and writing manuscript. DM, MT, MF, and SP labs participated in the study and editing manuscript. PJ, EP, and EB performed the experiments and edited manuscript. RS participated in writing manuscript.

## Conflict of Interest

The authors declare that the research was conducted in the absence of any commercial or financial relationships that could be construed as a potential conflict of interest.

## Abbreviations

7-AAD: 7-aminoactinomycin D
APC: allophycocyanin
Best: Biomedical Excellence in Safer Transfusion
BM: bone marrow
CFU-F: colony forming unit-fibroblast
FBS: fetal bovine serum
FITC: fluorescein isothiocyanate
GvHD: graft-versus-host disease
hPL: human platelet lysate
LN2: liquid nitrogen
MLR: mixed lymphocyte reaction
MNC: mononuclear cell
MSC: mesenchymal stromal cell
P#: passage number
PCA: principal component analysis
PD: population doubling
PE: phycoerythrin
RCT: randomized control trials.
